# Comparative Study of Chemical Stability of a PK20 Opioid–Neurotensin Hybrid Peptide and Its Analogue [Ile^9^]PK20—The Effect of Isomerism of a Single Amino Acid

**DOI:** 10.3390/ijms231810839

**Published:** 2022-09-16

**Authors:** Barbara Żyżyńska-Granica, Adriano Mollica, Azzurra Stefanucci, Sebastian Granica, Patrycja Kleczkowska

**Affiliations:** 1 Chair and Department of Biochemistry, Faculty of Medicine, Medical University of Warsaw, 02-097 Warsaw, Poland; 2 Department of Pharmacy, G. d’Annunzio University of Chieti-Pescara, 66100 Chieti, Italy; 3 Microbiota Lab, Department of Pharmacognosy and Molecular Basis of Phytotherapy, Medical University of Warsaw, 02-097 Warsaw, Poland; 4 Military Institute of Hygiene and Epidemiology, 01-163 Warsaw, Poland; 5 Maria Sklodowska-Curie Medical Academy in Warsaw, 03-411 Warsaw, Poland

**Keywords:** hybrid peptides, stability, degradation, chemical structure

## Abstract

Chemical stability is one of the main problems during the discovery and development of potent drugs. When ignored, it may lead to unreliable biological and pharmacokinetics data, especially regarding the degradation of products’ possible toxicity. Recently, two biologically active drug candidates were presented that combine both opioid and neurotensin pharmacophores in one entity, thus generating a hybrid compound. Importantly, these chimeras are structurally similar except for an amino acid change at position 9 of the peptide chain. In fact, isoleucine (C_6_H_13_NO_2_) was replaced with its isomer *tert*-leucine. These may further lead to various differences in hybrids’ behavior under specific conditions (temperature, UV, oxidative, acid/base environment). Therefore, the purpose of the study is to assess and compare the chemical stability of two hybrid peptides that differ in nature by way of one amino acid (*tert*-leucine vs. isoleucine). The obtained results indicate that, opposite to biological activity, the substitution of *tert*-leucine into isoleucine did not substantially influence the compound’s chemical stability. In fact, neither hydrolysis under alkaline and acidic conditions nor oxidative degradation resulted in spectacular differences between the two compounds—although the number of potential degradation products increased, particularly under acidic pH. However, such a modification significantly reduced the compound’s half-life from 204.4 h (for PK20 exposed to 1M HCl) to 117.7 h for [Ile^9^]PK20.

## 1. Introduction

Drug degradation, either enzymatic or chemical, is crucial for its clinical response and efficacy. However, most drugs available tend to have a short half-life or are unstable as a result of their exposure to various environmental factors. In addition to enzymatic stability, the chemical stability of the molecule is another equally important feature of all compounds tested for potential medical use. It determines the sensitivity of the compound to degradation by various non-enzymatic processes. In fact, several chemical reactions may affect structures in aqueous solutions, including hydrolysis, deamidation, isomerization or oxidation [1]. Each of these processes may lead to the transformation of the physicochemical properties of the compound, which may occasionally be manifested by a change in the action profile and therapeutic potential.

The most important factors that can often cause inactivation of drugs are temperature (both too low and too high), environmental pH and UV radiation. This property is important due to the exposure of peptide drugs to unfavorable conditions during the production process and subsequent storage and use of the finished preparation. Furthermore, the resistance of the drug substance to the degrading effect of acidic pH is important in the case of oral formulations that enter the stomach. High chemical stability is necessary for the drug substance to exhibit adequate biological activity throughout the shelf-life of the preparation and to be able to produce desired effects. Indeed, as the drug undergoes degradation, it becomes less effective. In addition, drug decomposition may yield toxic byproducts that are harmful to the patient.

All of the abovementioned factors are especially true for peptide compounds, reducing their therapeutic application. In fact, peptides, compared to other structures formed by antibodies or proteins, are more susceptible to enzymatic and chemical degradation. They owe this property to their chemical structure, as each amino acid in the sequence is linked to the other by way of peptide bonds (amide bonds), which undergo spontaneous degradation through hydrolysis. Other reactions may occur, such as diketopiperazine formation as a consequence of the degradation of the *N*-terminus [2,3]. In addition, oxidation appears to be the most important type of chemical degradation of protein/peptides, which can be affected by pH [4] or even the flexibility of the peptide backbone [5].

**Figure 1:**
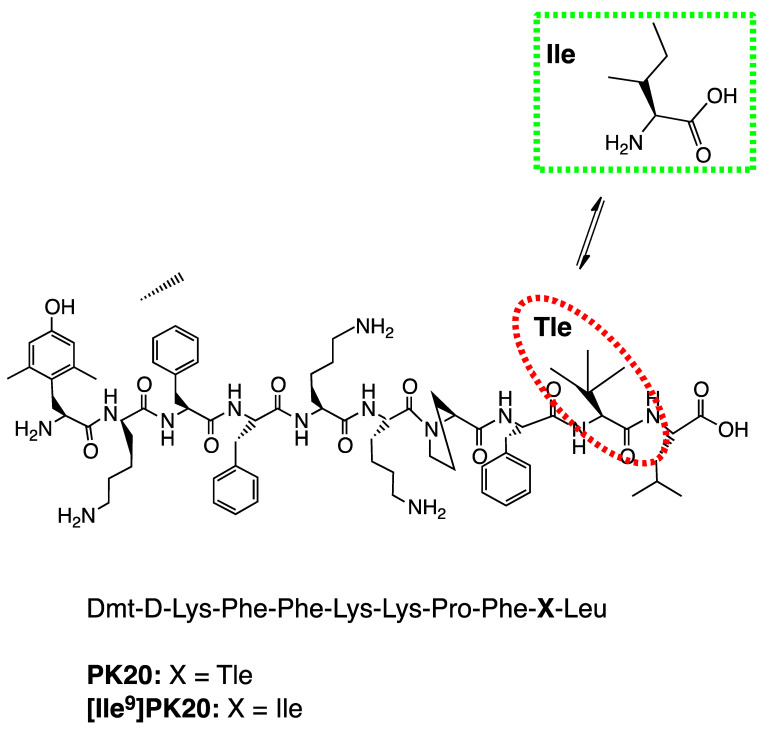
Chemical structure of the PK20 opioid–neurotensin hybrid peptide with the indication of a performed modification resulting in the production of [Ile^9^]PK20.

Since hybrid compounds have gained attention due to their potent multifunctional behavior with a more favorable profile in terms of possible side effects, much work devoted to such compounds can be found. PK20 and [Ile^9^]PK20, which are hybrid structures encompassing both opioid and neurotensin pharmacophores, although modified, were designed and synthesized to reduce pain. Indeed, a modified pharmacophore of an opioid endorphin-2 (Tyr-Pro-Phe-Phe-NH₂) has been designed by an incorporation of known elements that possess the ability to increase its resistance to metabolic degradation: Tyr was replaced by Dmt in position 1 and a *D*-amino acid residue (*D*-Lys) was inserted in position 2 of the peptide sequence. In addition, a neurotensin pharmacophore (*pyr*Glu-Leu-Tyr-Glu-Asn-Lys-Pro-Arg^8^-Arg^9^-Pro-Tyr-Ile-Leu-OH) was strongly reduced and modified, especially by the replacement of Arg8-Arg9 with Lys-Lys. Nonetheless, the structures of these two hybrid peptides are similar (Figure 1), if not the same, except for one amino acid: *tert*-leucine → isoleucine (Tle → Ile). An additional substitution such as Ile12 by Tle was suggested to improve the metabolic stability [6]; PK20 revealed its resistance to degradation as the exact half-life of the peptide was calculated to be 31 h 45 min [7]. However, most of the drug biological activity was affected, which was further confirmed in our studies focused on antinociceptive effect [8,9] and neuroprotective effects. For example, [Ile^9^]PK20-induced analgesia was significantly lower: 0.005 at 120 min and 0.02 nmol/rat after specific time-points of 30, 60 and 120 min, respectively, when compared to PK20. PK20 was also significantly stronger than its structural analogue at a dose of 0.02 nmol/rat and at 30 min drug post-administration when compared to morphine (3 nmol/rat) [8,9]. These were also confirmed by differences in the intrinsic activity of the receptor which was targeted ([Ile^9^]PK20 had lower efficacy (Emax = 151.2% ± 74.5) and potency at mu opioid receptor (EC50 = 1244 nM) relative to PK20 (Emax = 149.17% ± 2.9 and EC50 = 79 nM), respectively) [9]. Additionally, the protective properties of PK20 and [Ile^9^]PK20, assessed in an in vitro model of excitotoxic injury in organotypic hippocampal slice cultures subjected to NMDA, demonstrated PK20 hybrid as a more potent agent when compared to its [Ile^9^]-analogue. The extent of damage to the CA1 region of the hippocampus, in the case of a combined administration of PK20 and NMDA, was equal: 1.47% ± 1.20 for the dose of 25 ng/mL, 5.18% ± 2.84 for the dose of 50 ng/mL and 6.82% ± 6.08 for the dose of 100 ng/mL, respectively [10]. In contrast, for [Ile^9^]PK20 administered simultaneously with NMDA, the values were as follows: 8.53% ± 2.65 for the dose of 25 ng/mL, 19.04% ± 8.52 for the dose of 50 ng/mL and 8.62% ± 0.62 for the dose of 100 ng/mL, respectively (data not published).

Tle and Ile are structural isomers belonging to the same class of leucines with the same atomic composition (C_6_H_13_NO_2_) but they have different chemical structures, including different bond coordination and stereochemistry (Figure 1). Thus, their presence in the peptide structure led to diverse results. In fact, incorporation of Ile into the 9th position of the peptide chain caused a decrease in the analgesic activity in vivo when compared with PK20, having a tert-leucine (Tle9) in the corresponding position [8]. Nonetheless, [Ile^9^]PK20 produced the maximal pain-relieving effect, although with a distinct pharmacokinetic profile in comparison with PK20 [9]. Furthermore, in the case of [Ile^9^]PK20, a replacement of Tle with Ile led to reduced binding at the target receptors (i.e., mu opioid receptor and NTS1 neurotensin receptor) [9].

Considering the aforementioned, this paper aims to present whether such a slight structural difference may influence the chemical stability of both drugs when exposed to thermal, acidic/basic, oxidative and UV factors. 

## 2. Results

### PK20 and [Ile^9^]PK20 Degradation Depending on Conditions

**Figure 2:**
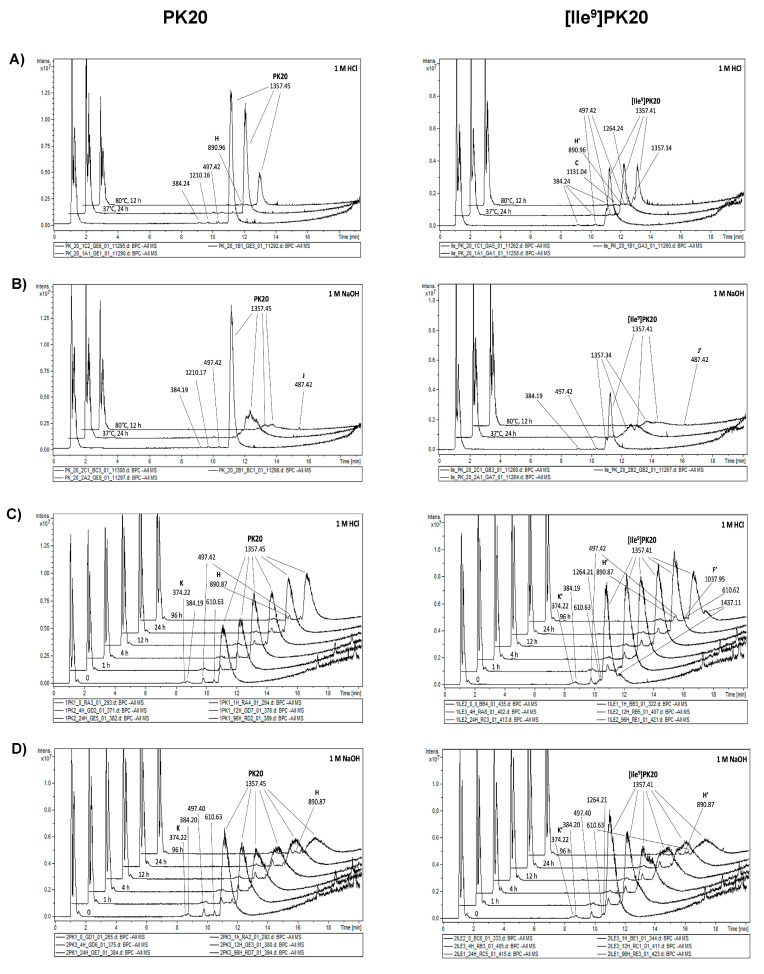
PK20 (panel left) and [Ile^9^]PK20 (panel right) degradation in acidic (1M HCl) and basic (1M NaOH) conditions depends on: the temperature and time of exposure (panel (**A**) and (**B**), respectively) and time of exposure (panel (**C**) and (**D**), respectively). Chromatograms show degradation products with corresponding molecular weights.

**Figure 3:**
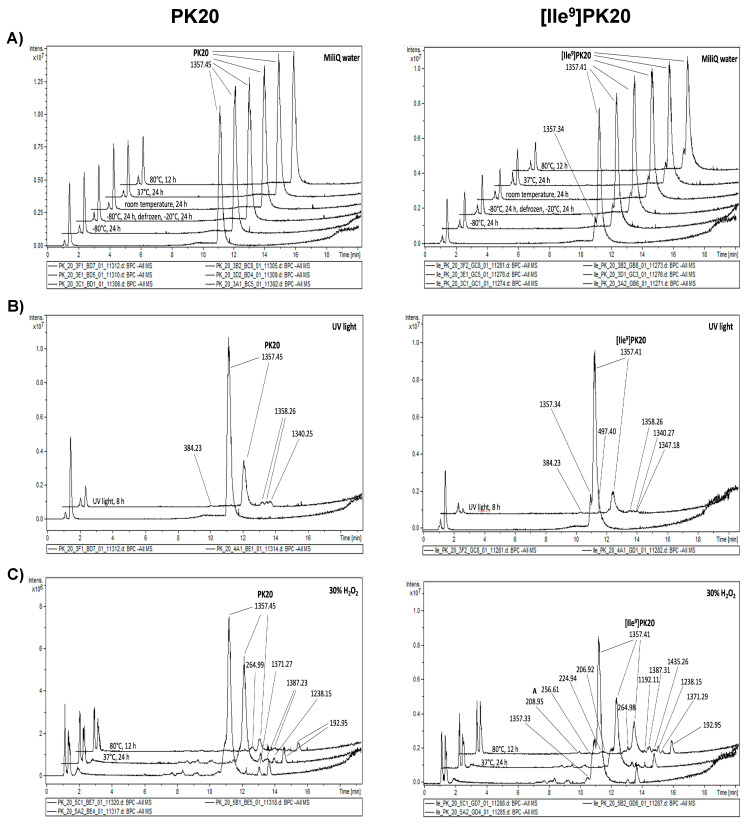
Thermal (**A**) and photolytic (**B**), and oxidative (**C**) PK20 (panel left) and [Ile^9^]PK20 (panel right) chimera degradation.

PK20 opioid–neurotensin hybrid peptide was found to be quite stable, particularly in thermal and acidic degradation (Table 1, Figure 2A and Figure 3A—left panel). At varying temperatures ranging from 22 °C (room temperature) through 37 °C to +80 °C, the remaining concentration of the peptide was approximately 50 μg/mL, which corresponds to 100%. Furthermore, PK20 behaved similarly when incubated at room temperature or −80 °C (Table 1), resulting in a 100% recovery. In contrast, alkaline stress led to significant peptide degradation (Table 1, Figure 2—left panel). In fact, after 24 h of hydrolysis in 1 M NaOH and at 37 °C, the percentage of PK20 remaining was 30.32%, while after 24 h of hydrolysis in 1M hydrochloric acid, the recovery reached almost 80% (78.19%). In addition, the calculated half-life of PK20 in 1M HCl was 204.4 h, while in 1M NaOH, t_1/2_ was 11.36 h.

**Figure 4:**
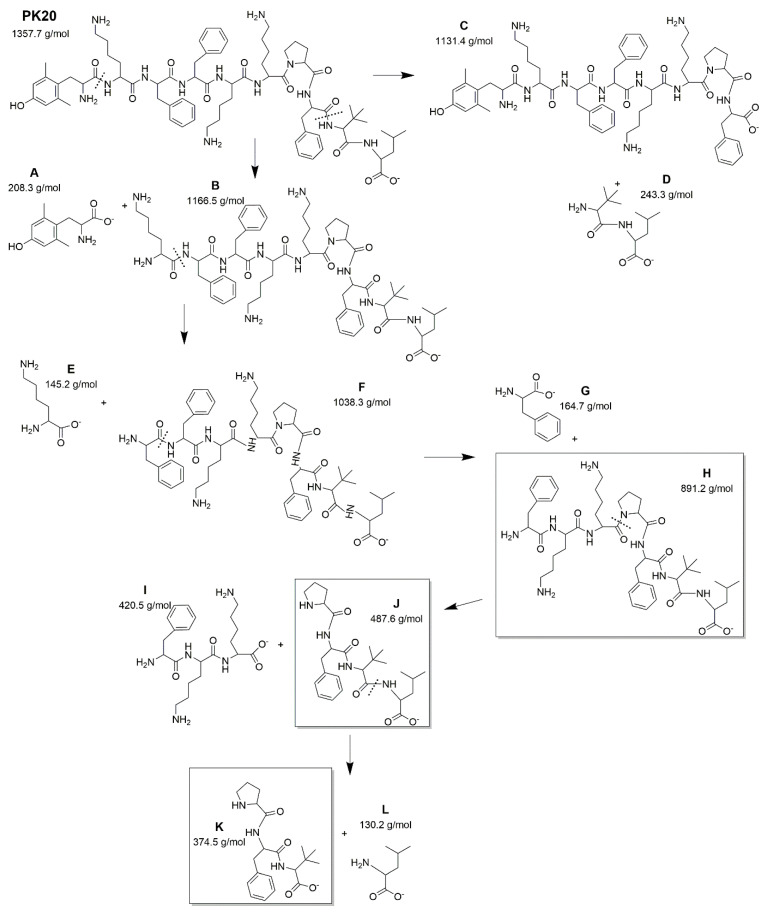
Representative structures of potential PK20′s degradation products based on the molecular weight obtained. Products commonly observed in Figure 2 are given in brackets, and these are as follows: compound K—Pro-Phe-Tle [m/z observed = 374.22 g/mol vs. MW calculated = 374.5 g/mol]; compound J—Pro-Phe-Tle-Leu m/z observed = 487.42 g/mol vs. MW calculated = 487.6 g/mol]; compound H—Phe-Lys-Lys-Pro-Phe-Tle-Leu [m/z observed = 890.96 g/mol vs. MW calculated = 891.2 g/mol] with PK20 [m/z observed = 1357.45 g/mol vs. MW calculated = 1357.7 g/mol].

Importantly, neither acidic nor basic degradation provided at the higher temperature of +80 °C for 12 h showed any of PK20′s stability improvement; the percentage of PK20 remaining was 54.43 and 11.44%, respectively. It is noteworthy that, in all of the studied stress conditions, PK20 degraded to similar degradants, as it was revealed in Figure 2A,B (left panel). However, one of the newly produced compounds was characteristic for alkaline conditions only: Compound J (Figure 4) with a corresponding m/z of 487.42 (Figure 2B; left panel).

The replacement of Tle in PK20 with Ile in [Ile^9^]PK20 did not significantly change the compound’s chemical stability. In fact, neither hydrolysis under alkaline and acidic conditions nor oxidative degradation resulted in spectacular differences between the two compounds–although the number of potential degradation products increased, particularly under acidic pH (Figure 2A,C; right panel). Interestingly, only the [Ile^9^]PK20 exposure to UV light led to an observable decrease in the stability, as the percentage of the compound remaining was only 19.25% (in comparison with 37.44% for PK20) (Table 1). Some additional differences were noted when the compound was treated with temperature, especially under the two freeze–thaw cycles. Surprisingly, [Ile^9^]PK20 was much more resistant than its mature hybrid compound PK20, as the recovery reached approximately 100% (Table 1). 

Even though there were no changes in the recovery values for both compounds examined under acidic and basic conditions, the [Ile^9^]PK20 half-life deteriorated significantly, estimated at 117.7 h for acidic and 4.69 h for alkaline conditions.

The exposure of an aqueous solution of PK20 to UV light for 7 h resulted in a slight degradation (Table 1, Figure 3B—left panel). A similar result was found for PK20 under oxidative stress (Table 1, Figure 3C—left panel), although an increase in temperature worsened the obtained results. For PK20 treatment with 30% H_2_O_2,_ either at a temperature of 37 °C for 24 h or 80 °C for 12 h, the remaining compound was 61.04 and 19.60%, respectively (Table 1). Importantly, some novel degradants were detected as a result of H_2_O_2_ treatment, with molecular weights exceeding the value of PK20 (m/z = 1357.45 g/mol) (Figure 3C; left panel). This was true for the peaks with the corresponding m/z of 1371.27 and 1387.23 g/mol.

As was presented in Figure 2, the PK20 chimera is stable under specific conditions, although some degradation products can be distinguished (Figure 4). According to the time of appearance on the chromatogram (retention time), one of the first degradation products (T_R_ = 8.6 min) was the compound **K** with m/z of 374.22 g/mol corresponding with the amino acid structure Pro-Phe-Tle (Figure 2C,D (left panel) and Figure 4). In both acidic and basic stress, PK20 degradation resulted in the formation of a compound **H** with a molecular mass of 890.96 g/mol (T_R_ = 10.0 min) (Figure 2; left panel). Furthermore, in both conditions, PK20 was degraded to unknown products and/or impurities at the retention time of PK20 peak (11.2 min): 8.6 (MW = 374.22 g/mol), 9.2 (MW = 384.24 g/mol), 9.7 (MW = 1210.16 g/mol) and 10.6 min (MW = 497.40 g/mol). However, only under alkaline stress, at 80 °C, and at the time-point of 13.6 min, did the chimera produce the compound **J** (Pro-Phe-Tle-Leu, MW = 487.42 g/mol) (Figure 2B (left panel) and Figure 4).

Although both PK20 and its analogue [Ile^9^]PK20 are quite similar in terms of their stability under thermal, photolytic and oxidative stress (Figure 3, Table 1), where the differences occurred. These relate to the quantity and quality of the degradation products. Indeed, as it was presented, the hybrid peptide [Ile^9^]PK20 breaks down into a much larger amount of degradation products (Figure 3—right panel)—though some are the same for PK20 (e.g., compounds with m/z of 1340 and 384 for UV light exposure; Figure 3B, right and left panels). 

**Figure 5:**
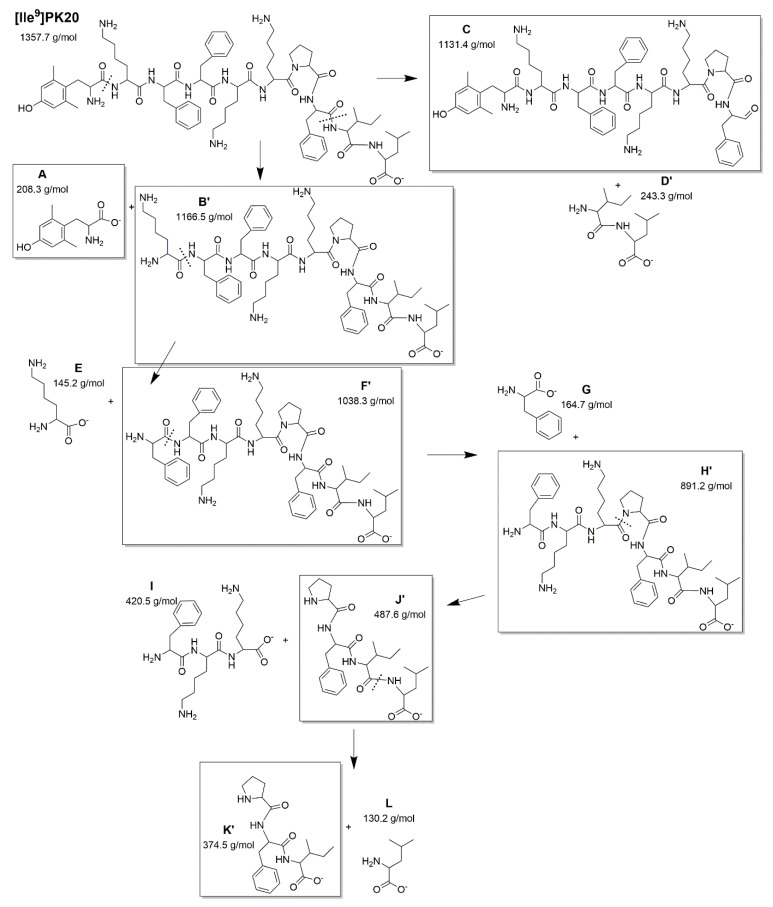
Representative structures of potential [Ile^9^]PK20’s degradation products based on the molecular weight obtained, and identified as: compound K’—Pro-Phe-Tle [m/z observed = 374.22 g/mol vs. MW calculated = 374.5 g/mol]; compound J’—Pro-Phe-Tle-Leu [m/z observed = 487.42 g/mol vs. MW calculated = 487.6 g/mol]; compound H’—Phe-Lys-Lys-Pro-Phe-Tle-Leu [m/z observed = 890.96 g/mol vs. MW calculated = 891.2 g/mol] with [Ile^9^]PK20 [m/z observed = 1357.45 g/mol vs. MW calculated = 1357.7 g/mol].

Possible structures of several new degradants are presented in Figure 5.

## 3. Discussion 

Peptides are known for their extreme instability under enzymatic and chemical conditions, which are particularly important in terms of drug storage and the route of its administration into the body. This, in turn, is critically important to both the safety and efficacy of drugs. Such stability, or lack thereof, is due to the structure of the molecule. The prediction of the possible pathway of degradation enables an understanding of labile functionalities crucial in designing less reactive and more stable analogues [11]. Therefore, herein we decided to determine whether substituting one amino acid with its structural isomer may influence the compound stability when exposed to different stress conditions. 

Based on previous studies demonstrating [Ile^9^]PK20 as a much weaker analgesic peptide in comparison to PK20, with a completely different pharmacokinetic profile [8,9] it was probable that the insertion of Ile instead of Tle into the compound’s peptide chain could also result in dramatic changes and dissimilarities in chemical stability. However, none of the factors used, such as acidic or basic pH, oxidative conditions and low or high temperature, resulted in substantial changes between either peptide. Moreover, these two compounds were found to be stable, particularly under acidic conditions. Additionally, thermal treatment did not affect the recovery of the peptides. However, when exposed to UV light for 7 h, [Ile^9^]PK20 turned out to be more rapidly degraded than its mature compound, as the percentage of [Ile^9^]PK20 remaining was only 19.25% (in comparison with 37.44% for PK20). 

Although there were no important changes in the behavior of both compounds, the replacement of Tle with Ile in the peptide chain influenced their half-life. 

The half-life is a key element in the therapeutic efficacy and potency of drug molecules at the site where it is administered. In addition, it is well known that incorporating a non-natural amino acid into the peptide results in the extension of its half-life [12]. This is consistent with our results demonstrating Tle as a crucial element that possibly induces conformational changes of the entire molecule. Thus, the final effect is the improvement in the resistance to the action of numerous stress factors. Indeed, the presence of Tle in PK20 led to the achievement of long-term stability, both in acidic and basic conditions. The PK20 half-life values were 204.4 h for 1 M HCl and 11.36 h for 1 M NaOH. At the same time, Tle substitution for Ile resulted in a significant reduction in the half-life: 117.7 h for acidic and 4.69 h for alkaline conditions [13].

When analyzing the mass peaks from the LC-MS/MS and, further, the type of the assigned structure that were formed under the stress conditions, significantly more peaks were shown for [Ile^9^]PK20 compared with PK20. The degradation reactions, and ultimately the efficiency of degradation processes, vary as a function of the environmental conditions and reaction types, and are dependent on the structure of the compound exposed. In line with this, apart from the identified degradation products, the used factors (i.e., UV, temperature, acidic or basic pH, etc.) possibly induced modifications on the amino acids, as some products with the molecular weight exceeding the output value were detected. The situations mentioned above are well known in the literature [14,15]. For example, as provided by Alsant et al. [11], changes in the MW of +16 and +32 amu occur frequently and correspond to the addition of one and two oxygen atoms, respectively. Likewise, a change in the MW of +18 or −18 amu can readily be explained by the addition or loss of water. Additionally, other products have been described in the literature, such as side chain ring opening or isobaric conversion between amino acids by way of the loss of side chain groups [16]. Furthermore, UV irritation is well known for its ability to produce reactive species, including hydroxyl radicals (OH^•^), superoxides (O_2_^−^), solvated free electrons (e_aq_^−^), hydroperoxyls (HO_2_^•^) and hydrogen peroxide (H_2_O_2_) [16,17]. Similarly, several free or bond amino acids can be found to undergo photolytic oxidation when exposed to UV light or H_2_O_2_ solely [18,19]. Our studies revealed numerous unidentified degradation products, although we can suspect the modification type from the change of m/z. For instance, the decrease in mass of −17 amu under UV light suggests detachment of the OH^•^ radical (1340.25). However, most of the degradation products cannot be identified only by LC-MS/MS analysis. Nonetheless, additional studies are needed to determine the exact structures of every potential degradation product or impurities, as well as to predict the pathway of degradation.

## 4. Materials and Methods

### 4.1. Drugs and Chemicals

PK20 (Dmt-D-Lys-Phe-Phe-Lys-Lys-Pro-Phe-Tle-Leu-OH) and its analogue [Ile^9^]PK20 (Dmt-D-Lys-Phe-Phe-Lys-Lys-Pro-Phe-Ile-Leu-OH) were synthesized as previously described using Fmoc-based solid-phase peptide synthesis (SPPS) [20]. Methanol (LC-MS grade) and acetonitrile (LC-MS grade) were purchased from Merck KGaA (Darmstadt, Germany). Deionized water was produced with a Simplicity UV system (Merck-Millipore (Burlington, MA, USA). Hydrogen peroxide (H_2_O_2_), sodium hydroxide (NaOH) and hydrochloric acid (HCl) were purchased from POCH (Gliwice, Poland). 

### 4.2. LC Apparatus and LC-MS Conditions

The LC-MS/MS was performed using a Dionex Ultimate 3000RS device (Dionex, Sunnyvale, CA, USA) equipped with an autosampler, column oven and degasser. The chromatograph was coupled with a Bruker Amazon SL ion trap mass spectrometer (Bruker Daltonik, Bremen, Germany) without splitting.

The separation was carried out using Kinetex XB-C_18_ column (150 mm × 2.1 mm × 1.7 μm; Phenomenex, Torrance, CA, USA). The mobile phase consisted of A: 0.1% formic acid in deionized water and B: 0.1% formic acid in acetonitrile (MeCN). The two-step gradient was used from 5% B to 50% B in 13 min and from 50% B to 65% B up to 20 min. The column temperature was maintained at 25 °C with a flow rate equal to 0.3 mL/min. The eluate was introduced directly to the mass spectrometer. The ion trap setting was as follows: capillary voltage 4500 V, endplate offset 500 V, nebulizer pressure 40 psi, drying gas temperature 145 °C and gas flow rate 9 L/min. The instrument used a smart parameter setting (SPS) fixed at 1000 amu. The scan range was from m/z = 70 to m/z = 2200. Compounds were analyzed in negative ion mode.

### 4.3. The Stock Solution of Investigated Compounds

Accurately weighed, around 1 mg of each compound was dissolved in deionized water to reach the stock solution’s concentration of 1 mg/mL. The stock solution was prepared before each experiment and used immediately. 

### 4.4. Quantification of Peptides Using LC-MS 

Before LC analysis, the ionization of each quantified compound in the positive ion mode was optimized by the direct injection of standard (50 µg/mL in 0.1% HCOOH in H_2_O; 0.3 mL/min) into the ESI source of the mass spectrometer. The intensity of the most abundant ion for each compound was monitored in order to choose optimal conditions. The quantification of investigated compounds was performed using the dominating ion (for both PK20 and [Ile^9^] PK20 the ion at *m/z* =1357.40 ± 0.3, retention time ca. 11.2 min).

The calibration curves were plotted as the amount of injected compound (ng) vs. detector response (peak area) using extracted ion chromatograms (EIC) for the characteristic ion for each compound. The linear range for each quantified compound was between 5 and 200 ng per injection. Five amount levels were used for the plotting of curves. Samples at each level were analyzed in triplicate. 

### 4.5. Method Validation

The method for quantifying peptides detected during the LC-MS analysis was validated according to ICH guidelines [21]. Method selectivity, sensitivity, linearity and precision were evaluated. The developed method met ICH criteria in each of the evaluated aspects. The method was selective, sensitive, linear and precise and could be used to assess the degradation processes of investigated peptides. Calibration curves, recorded MS spectra for standards, samples chromatogram of standards and method precision and recovery data are given in the supplementary materials (Figures S1–S3 and Table S1).

### 4.6. Degradation and Analysis Procedures of the Stressed Compounds 

The stability of PK20 and [Ile^9^]PK20 was tested under acidic, basic, oxidative, heat, freeze and ultraviolet light conditions. All experiments were conducted in triplicate (n = 3). The comparison of peak areas in degradative and non-degradative conditions for both compounds can be observed in Figure 3 and Figure 4. 

#### 4.6.1. Acid and Base Hydrolysis

Drug solution (50 μg/mL) was prepared in HCl (1 M) or NaOH (1 M). Aliquots were kept at a temperature of 80 °C (LBK type water bath, SWL Bytom, Poland) for 12 h and 37°C (medical water bath, LW102, Auritronic, Krakow, Poland) for 24 h. At a specific time-point, acidic and basic solutions of both PK20 and [Ile^9^]PK20 were neutralized with an equal volume of 1 M NaOH or 1 M HCl, respectively, and diluted with an equal volume of 0.2% formic acid in acetonitrile and analyzed using LC-MS.

#### 4.6.2. Thermal Degradation

Solutions (50 μg/mL) of both hybrid peptides were prepared in Mili-Q water, and aliquots were kept at room temperature 22 °C, 37 °C and −80 °C for 24 h and at 80 °C for 12 h. One aliquot was also subjected to two freeze–thaw cycles with the sample first frozen at −80°C for 24 h, defrosted under running tap water at room temperature for 2 h and second frozen at −20°C for 24 h and defrosted under running tap water at room temperature for 2 h. Then, the solutions of PK20 and [Ile^9^]PK20 were diluted with an equal volume of 0.2% formic acid in acetonitrile and analyzed with LC-MS.

#### 4.6.3. UV Degradation

PK20 and [Ile^9^]PK20 solution (50 μg/mL) was prepared in Mili-Q water, and aliquots were kept in clear plastic vials exposed to UV light (366 nm, Camag, UV Lamp 4, Camag, Switzerland) for 7 h. After the incubation, the drug solution was diluted with an equal volume of 0.2% formic acid in acetonitrile and analyzed with LC-MS.

#### 4.6.4. Oxidative Degradation

PK20 and [Ile^9^]PK20 solution (50 μg/mL) was prepared in 30% (*v*/*v*) hydrogen peroxide (H_2_O_2_). Aliquots were incubated at 37 °C for 24 h and 80 °C for 12 h. After the incubation, each drug solution was diluted with an equal volume of 0.2% formic acid in acetonitrile and analyzed with LC-MS.

## 5. Conclusions

Drug degradation is crucial for the clinical response of a compound. Here, we compared the chemical stability of two hybrid peptides that differ in nature by way of one amino acid (*tert*-leucine vs. isoleucine in PK20 and [Ile^9^]PK20, respectively). Our studies indicated that, although the difference in chemical stability is not substantial, the half-time of PK20 is significantly higher than for [Ile^9^]PK20 for both basic as well as acidic conditions. Moreover, the recovery of PK20 is higher for both UV and oxidative conditions. These differences may be important for future in vivo use of both compounds. Additionally, our study shows, for the first time, that the simple change of one amino acid can influence the chemical stability of a whole molecule. Thus, it is important to perform stability analysis for every analogue of a known compound, even if the modification is small and seems to be negligible.

## Figures and Tables

**Table 1:** Summarized results obtained for the chemical degradation of PK20 and [Ile^9^]PK20 opioid–neurotensin hybrid peptides.

Condition	Drug Concentration Injected (μg/mL)	Concentration Found (Mean ± SD, μg/mL)		Recovery (%)		RSD (%) *	
		PK20	[Ile^9^]PK20	PK20	[Ile^9^]PK20	PK20	[Ile^9^]PK20
**Acidic degradation (1 M HCl)**							
37 °C (24 h)	50	39.09 ± 6.33	44.39 ± 4.48	78.19	88.79	16.18	10.10
80 °C (12 h)	50	27.22 ± 2.75	26.12 ± 1.52	54.43	52.25	10.09	5.83
**Basic degradation (1 M NaOH)**							
37 °C (24 h)	50	15.16 ± 0.34	18.56 ± 0.59	30.32	37.13	2.27	3.19
80 °C (12 h)	50	5.71 ± 1.67	5.61 ± 1.83	11.44	11.23	29.17	32.69
**Oxidative degradation (30% H_2_O_2_)**							
37 °C (24 h)	50	30.52 ± 0.49	31.39 ± 0.27	61.04	62.79	1.60	0.87
80 °C (12 h)	50	9.80 ± 0.2	8.24 ± 6.69	19.60	16.48	2.07	81.14
**Thermal degradation**							
Room temperature (24 h)	50	51.36 ± 1.64	49.71 ± 2.24	102.72	99.41	3.19	4.50
37 °C (24 h)	50	49.98 ± 0.35	44.16 ± 0.99	99.97	88.32	0.70	2.24
80 °C (12 h)	50	48.67 ± 2.74	43.27 ± 1.18	97.34	86.54	5.63	2.73
−80 °C (24 h)	50	51.90 ± 0.20	51.87 ± 0.40	103.80	103.74	0.38	0.77
Two freeze–thaw cycles (24 h (−80 °C), –2 h (22 °C), 24 h (−20 °C))	50	43.25 ± 9.42	49.04 ± 1.03	86.50	98.08	21.78	2.09
**Photolytic degradation (365 nm UV light 7 h)**	50	18.72 ± 0.94	9.63 ± 0.11	37.44	19.25	5.04	1.14

* RSD—relative standard deviation.

## Data Availability

All data in this article is available from the corresponding author upon reasonable request.

## Supplementary Materials

The supporting information can be downloaded at: https://www.mdpi.com/article/10.3390/ijms231810839/s1.
