# Supplementary material for: Comparative Study of Chemical Stability of a PK20 Opioid–Neurotensin Hybrid Peptide and Its Analogue [Ile9]PK20—The Effect of Isomerism of a Single Amino Acid
Source: Int J Mol Sci. 2022 Sep 16;23(18):10839. doi: 10.3390/ijms231810839 (PMC9500858; doi:10.3390/ijms231810839)
Supplement: Supplementary file 1 [file ijms-23-10839-s001.zip › ijms-1908173-supplementary.pdf]

Figure S1. EIC for PK20 and [Ile^9]PK20 generated for *m/z* = 1357.40 ± 0.20 showing peak of quantified compounds.

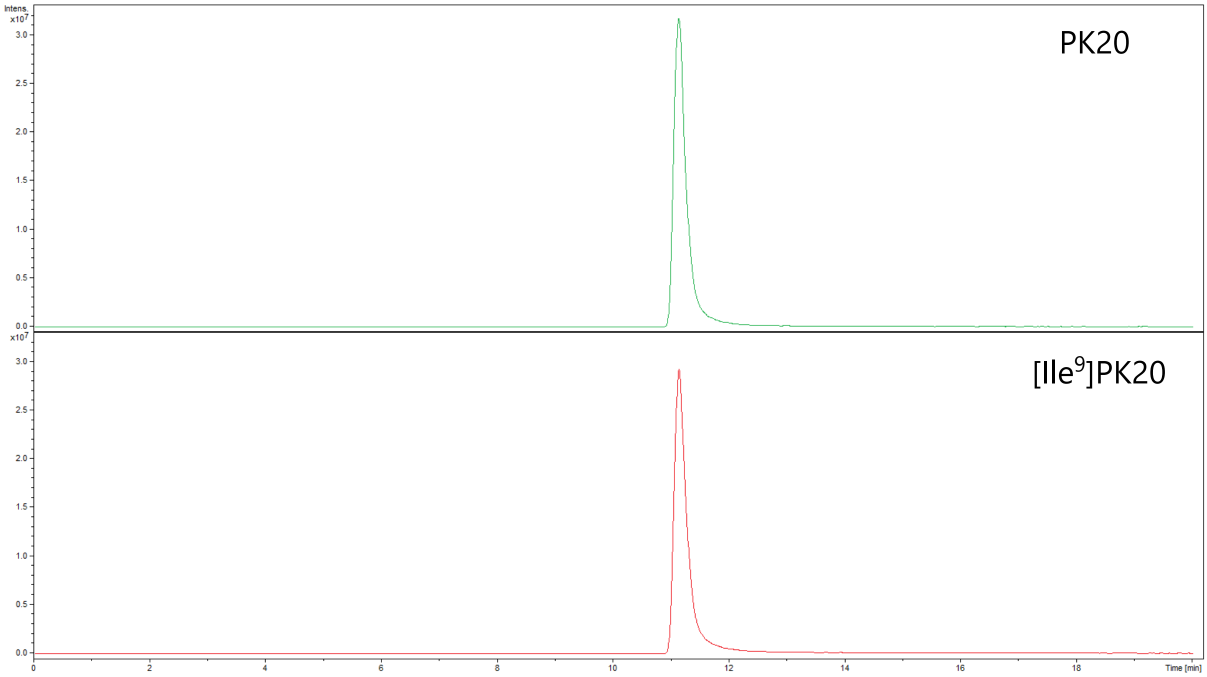

Figure S2. MS spectra recorded for PK20 (A) and [Ile^9]PK20 (B).

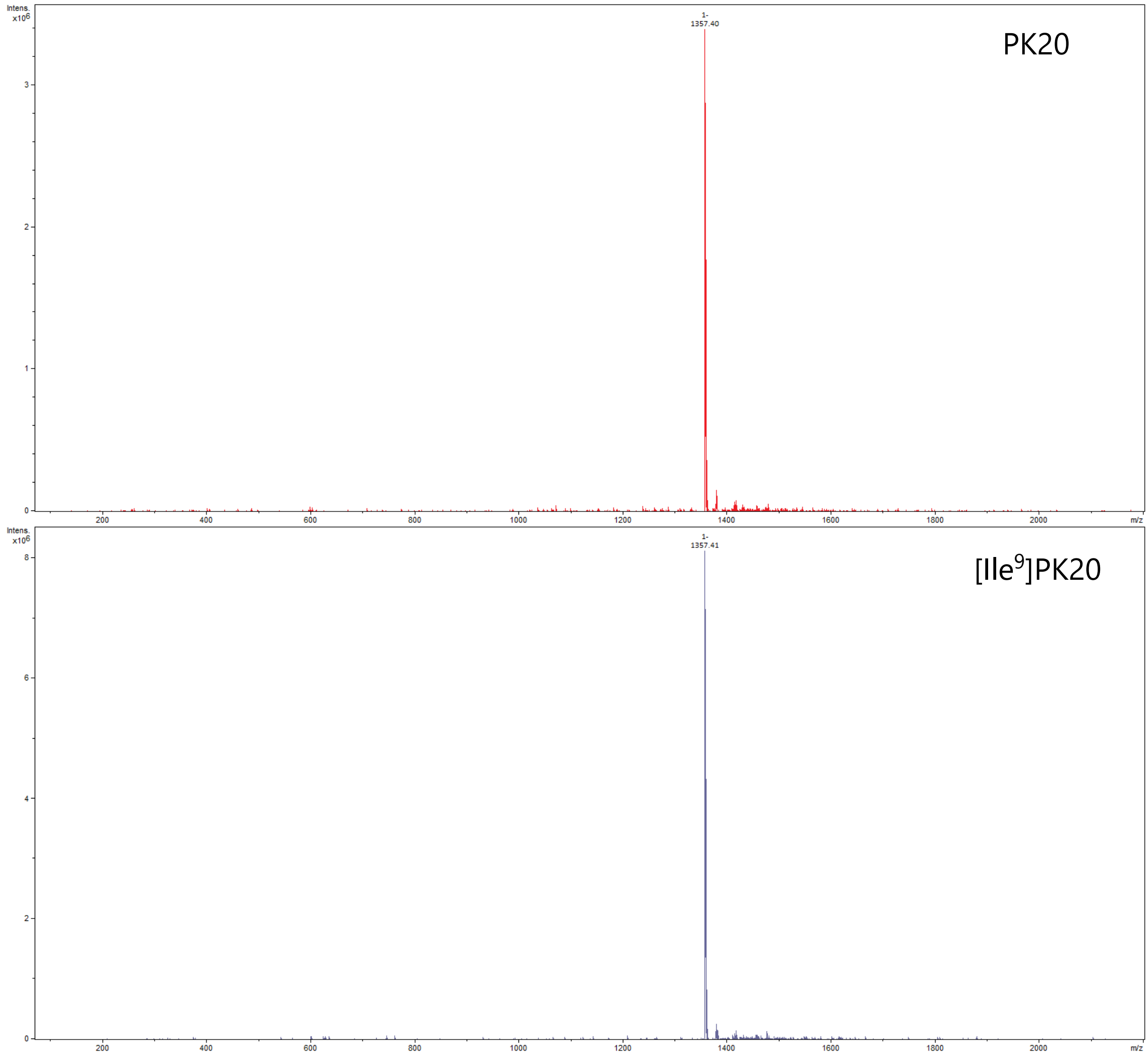

Figure S3. Calibration curves obtained for PK20 and [Ile^9]PK20 as area for EIC (*m/z* = 1357.40 ± 0.20) vs. ng of the peptide injected to LC column.

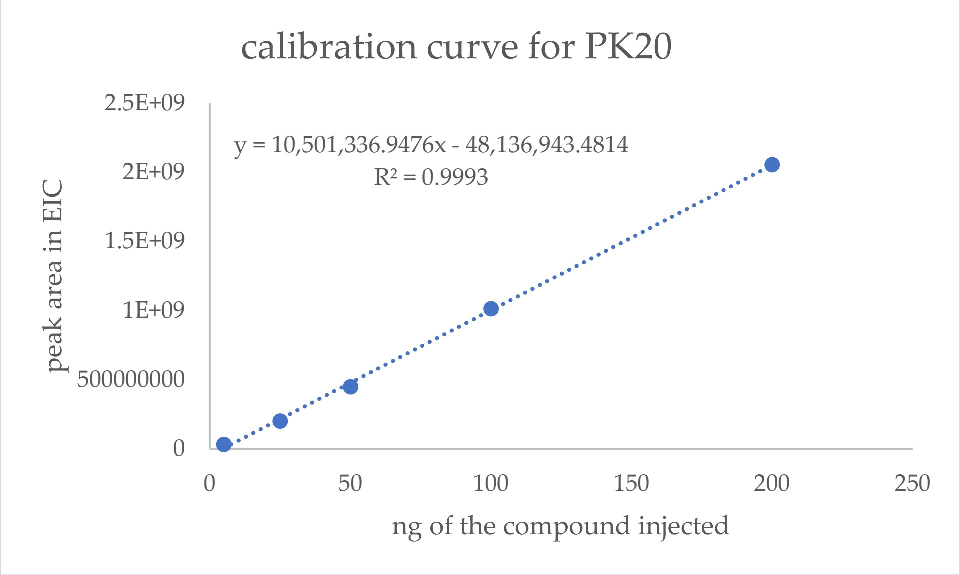

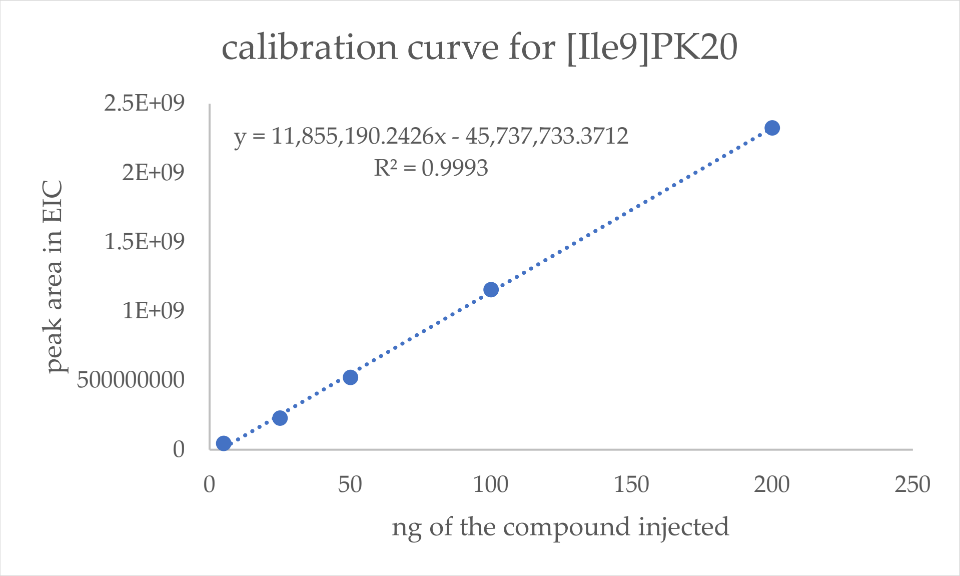

Table S1. Calibration and standard validation data for PK20 and [Ile^9]PK20.

Compound	Linear range /ng per injection	Calibration curve equation	Amount injected /ng	Intermidiate precison /%CV	Recovery /%	LOD /ng per injection	LOQ /ng per injection
PK20	5-200	y = 10220837.313x – 32897298.173	5	3.63	96.4 ± 0.7	2.92	8.84
			25	3.17			
			50	4.27	102.1 ± 0.6		
			100	4.34			
			200	1.73	101.2 ± 0.5		
[Ile^9]PK20	5-200	y = 11633076.337x – 33670205.577	5	1.79	95.6 ± 0.4	2.54	7.70
			25	2.60			
			50	0.77	99.4 ± 0.7		
			100	2.12			
			200	2.77	101.3 ± 0.3		
